# Prognostic value and co-expression patterns of metabolic pathways in cancers

**DOI:** 10.1186/s12864-020-07251-0

**Published:** 2020-12-29

**Authors:** Dan Zhang, Yan Guo, Ni Xie

**Affiliations:** 1 Biobank, Shenzhen Second People’s Hospital, The First Affiliated Hospital of Shenzhen University, Health Science Center, Shenzhen, 518035 China; 2 Comprehensive Cancer Center, University of New Mexico, Albuquerque, 87131 USA

**Keywords:** Metabolic pathway, Meta co-expression analysis, Gene expression composite score, Cancer

## Abstract

**Background:**

Abnormal metabolic pathways have been considered as one of the hallmarks of cancer. While numerous metabolic pathways have been studied in various cancers, the direct link between metabolic pathway gene expression and cancer prognosis has not been established.

**Results:**

Using two recently developed bioinformatics analysis methods, we evaluated the prognosis potential of metabolic pathway expression and tumor-vs-normal dysregulations for up to 29 metabolic pathways in 33 cancer types. Results show that increased metabolic gene expression within tumors corresponds to poor cancer prognosis. Meta differential co-expression analysis identified four metabolic pathways with significant global co-expression network disturbance between tumor and normal samples. Differential expression analysis of metabolic pathways also demonstrated strong gene expression disturbance between paired tumor and normal samples.

**Conclusion:**

Taken together, these results strongly suggested that metabolic pathway gene expressions are disturbed after tumorigenesis. Within tumors, many metabolic pathways are upregulated for tumor cells to activate corresponding metabolisms to sustain the required energy for cell division.

**Supplementary Information:**

The online version contains supplementary material available at 10.1186/s12864-020-07251-0.

## Background

Gene expression and metabolism are two essential biological processes critical to all living organisms. Gene expression is the fundamental information flow that transforms the heritable genetic information of individual genes to cellular functioning entities, including constituent proteins and catalytic enzymes. Metabolism refers to the system of all chemical reactions that are synergistically inter-connected to fulfill all respects of viability necessities. Abnormal metabolism has been added to the original six hallmarks of cancer in 2011 [1]. Harmonized gene expression and metabolic state are prerequisites for homeostasis - the maintenance of steady internal physical and chemical conditions in living systems. It has been well documented that there are close associations and regulatory effects between gene expression and metabolic state. Numerous models were developed to capture these associations between gene expression and metabolism [2, 3]. Most recently, it was shown that lactate-derived lactylation of histone lysine residues can directly stimulate gene transcription from chromatin [4]. This finding further proves that there is a strong metabolic regulation of gene expression through histone acylation [5]. Metabolic enzymes can serve as the link between metabolism and gene regulation [6].

A metabolic pathway consists of a cascade of enzyme-catalyzed chemical reactions occurring in a cell that are orchestrated to fulfill one relatively independent cellular function. In a metabolic pathway, the product of one upstream reaction acts as the substrate for the successive reaction, and all those substrates/products are termed metabolites in general. Many of the metabolites in these pathways have significant implications in cancer. For example, folate exerts its effect on cancer through nutrient-gene interaction as to genes within the folate metabolic pathway. Deficiency of folate has been linked to increased risk of cancers [7], and a clinical trial has shown the beneficial effect in malignant pleural mesothelioma [8]. Another well-studied metabolite is vitamin D, which has been shown to decrease cancer risks yet with some variability [9]. A plethora of metabolic pathways has been linked to cancer by supporting cell growth and proliferation through their effects on nutrient acquisition and lipid, protein, nucleic acids synthesis. For example, colon cancer cells that are deficient in p53, one of the most important tumor suppressors, activate the mevalonate pathway to adapt to the lack of oxygen and nutrients [10]. Many of the metabolic pathways’ functions can be activated by somatic mutations in oncogenes [11] or germlines variants [12]. It has been argued that tumor requires reprogrammed, more complex metabolism by loss of tumor suppressor or gain of oncogene, in order to promote cancer cell survival and growth [13].

Many metabolic pathways and corresponding metabolites have been thoroughly studied in cancers. These studies mostly focused on the cancer risk associated with nutrient supplement intake in the case-control type of epidemiology studies, or functional mechanism analysis of the regulatory effects between metabolism and gene expression. Previous pan-cancer metabolic studies [14–16] have shown that metabolic pathways have been significantly disturbed in cancer. In this study, we focus on the two aspects of cancer metabolism using two recently established bioinformatics methods. First, we evaluated whether metabolic pathway expressions have any predictability for cancer survival. Second, we examined the co-expression patterns within the metabolic pathways and differential expression of the metabolic pathways between paired tumor and normal samples.

## Methods

### Data acquisition

Twenty-nine major metabolic pathways curated by three sources: PID [17], PANTHER [18], and INOH [19] were extracted from Pathway Commons [20]. Pathways with identical names from distinct sources were merged into a single pathway by adopting the gene set with the largest size and adding additional gene members from a secondary pathway if that pathway contributed more than 70% shared gene members. Furthermore, we examined gene intersection between every pair of pathway and ensured each pair of pathways have no more than 70% common genes. The 29 metabolic pathways are abbreviated as MP1 to MP29 when necessary in this study. The full name of the metabolic pathways are listed in Supplementary Table S1.

### Survival analysis

The Cancer Genome Atlas (TCGA) data were downloaded from Genomic Data Commons for this study. Overall, processed RNA-seq data of 11,069 samples from 10,274 subjects of 33 cancer types were downloaded. Out of the 11,096 samples, 795 were adjacent normal tissues, and the rest were tumor tissues. The 33 cancer types alongside their abbreviations and sample sizes are listed in Supplementary Table S2. Detailed patient survival information including overall survival and disease specific survival were obtained from publication by Liu et al. [21]. Survival analyses were performed using Advanced Expression Survival Analysis (AESA) [22] from MutEx analysis suite [23]. AESA computed a composite gene expression score (CGES) for each of the 29 metabolic pathways within each cancer type. Each CGES was computed using the formula $CGES=\frac{1}{\left[1+\exp \left(-\left({\sum}_{i=1}^k{\beta}_i{x}_i-m\right)\right)\right]}$, where *x*_*i*_ denotes the standardized log-transformed expression value of the *i*th gene (from all *k* genes of a particular pathway) and the *β*_*i*_ denotes the score test coefficient for the same gene from a univariate Cox-proportional hazards regression. The normalization factor *m* captures the median of the linear combination term (${\sum}_{i=1}^k{\beta}_i{x}_i$) across all patients of the same cancer type, thus effectively normalizing the resultant CGES scores to the (0, 1) interval. Because CGES incorporates the coefficients from the expression-dependent Cox proportional hazards model and CGES is then put back into the same gene expression dataset, we applied a 1000-time permutation option offered by AESA to combat the effect of overfitting. The permutation *p*-value for each pathway-cancer scenario was adjusted for multiple tests using the Benjamini-Hochberg method. Adjusted p-value < 0.05 was considered statistically significant. ASEA has been previously used to successfully identify survival prediction values for non-coding RNAs [22].

### Meta co-expression analysis

Our second analysis of metabolic pathways focused on the expression difference between tumor and normal tissues. Two types of differences were interrogated: differential co-expression and differential expression. Differential co-expression analysis was assisted by Gene Sets Net Correlations Analysis (GSNCA) [24], which is a multivariate differential co-expression test that accounts for the complete correlation structure between genes of a pathway. The output of the original GSNCA method includes a permutation-derived *p*-value but is devoid of effect size and corresponding standard error. We adapted the GSNCA method into a meta analysis approach where multiple independent datasets can be used to determine the overall significance of co-expression changes between two conditions. To achieve this, we adapted a bootstrap approach to estimate the confidence interval. The effect size of an individual dataset can be estimated using the following formula: $ES={\mathit{\log}}_e\left(\frac{p}{1-p}\right)$, where p is the original GSNCA *p*-value. The meta analysis and forest plots were conducted using the generalized linear mixed effect model from R package Metafor [25]. In parallel to differential co-expression analysis, we conducted differential expression analysis between paired tumor and normal samples for the 29 metabolic pathways using paired t-test. Adjusted *p*-value < 0.05 is considered significant. The meta co-expression analysis has been built into a web application and can be accessed freely at http://www.innovebioinfo.com/Gene_Expression_Analysis/Meta_GSCA/MetaGSCA1.php.

## Results

### Survival analysis results

**Fig. 1:**
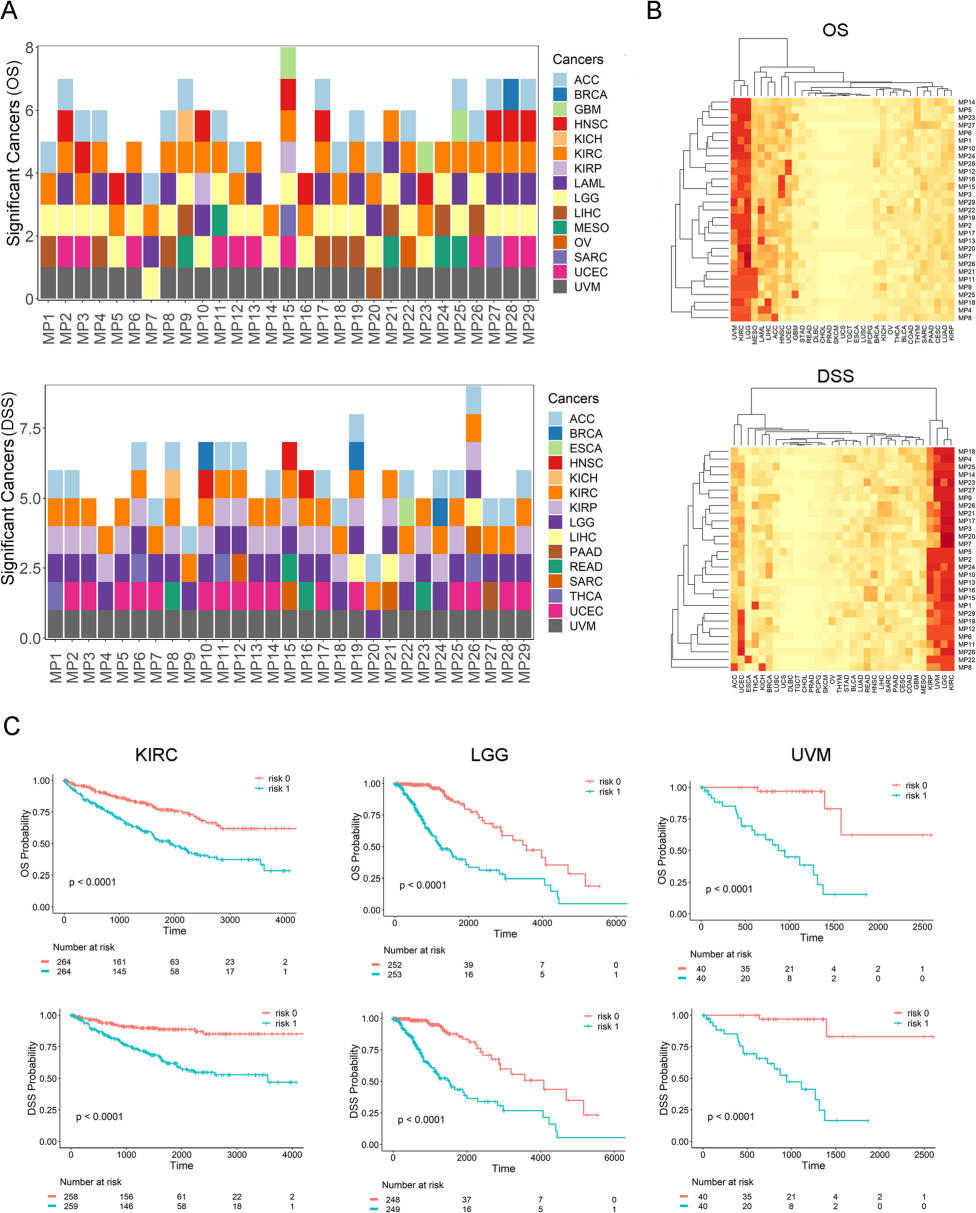
Overall and disease specific survival analysis results. **a**. Number of cancers with significant permutation *p*-value after multiple test correction for each metabolic pathways for overall survival and disease specific survival. **b**. Cluster analysis results based on overall survival and disease specific survival. Red color indicates more survival significance. **c**. Kaplan-Meier curves of folate metabolic pathway in three different cancers for both overall and disease specific survival. *P*-values labeled are the permutation *p*-values. Please see Supplementary Table S1 for metabolic pathway list (MP IDs) and Table S2 for cancer type list, respectively

Comprehensive survival analyses were conducted to evaluate the overall prognosis conferred by metabolic pathway expressions in cancers. Two types of survival data were obtained from publication by Liu et al. [21]: overall survival (OS) and disease specific survival (DSS). OS and DSS analyses were separately analyzed for each metabolic pathway within each cancer type. Each pathway was treated as a single entity by computing a CGES from AESA. Permutation of 1000 iteration was used to overcome overfitting. Of the 29 metabolic pathways tested in the 33 cancer types, the OS analyses affirmed statistical significance for 166 cancer-pathway combinations post permutation and multiple test correction (Supplementary Table S3), and the DSS analyses affirmed 170 similarly (Supplementary Table S4). The exact numbers of significant cancer types per pathway are displayed in Fig. 1a. On average, each metabolic pathway was significantly prognostic for OS of 5.7 cancer types (range: 3–8) or DSS of 5.9 cancer types (range: 3–9). MP15 (metabotropic glutamate receptor group II pathway) was significant in eight cancer types in OS analysis, and MP26 (pyruvate metabolism) was significant in nine cancer types in DSS analysis. Metabotropic glutamate receptors are widely known for synaptic signaling [26]. Recent evidence has suggested that glutamine can be used as an alternative energy source in place of glucose and serve as intermediates for macromolecule synthesis by tumor cells [27]. Pyruvate is another important metabolic compound and is a direct product of glucose metabolism. Pyruvate can directly induce the Warburg effect and it has been suggested to be a cancer therapeutic target [28].

Cluster analysis was conducted on the adjusted *p*-value with R package heatmap3 [29] using Euclidean distance (Fig. 1b). Explicit patterns can be seen from the cluster results. Metabolic pathway’s survival predictability is preferential to cancer types. Kidney renal papillary cell carcinoma (KIRC) and brain low grade glioma (LGG) have significant survival results for all 29 metabolic pathways, in both OS and DSS analyses. Uveal melanoma (UVM) affirmed statistical significance for 28 pathways in OS analysis and 27 in DSS analysis. Cancer types such as uterine corpus endometrial carcinoma (UCEC) and adrenocarotical carcinoma (ACC) had significant survival results for moderate numbers of metabolic pathways. Most other types of cancers had significant survival results for few or no metabolic pathways. For example, ovarian serous cystadenocarcinoma (OV) had significant survival result for one metabolic pathway in OS analysis and zero in DSS analysis.

Further exploring OS and DSS analysis results reveal that all significant survival results have the same direction. That is, increased overall gene expression level of a metabolic pathway always corresponds to worse survival outcome. Using MP5 (folate metabolism), an important cancer related metabolic pathway as an example, we plotted the Kaplan-Meier curves for cancer types KIRC, LGG and UVM (Fig. 1c) in both OS and DSS analyses. The universality of negative association between metabolic pathway gene expression and cancer survival clearly suggests that proliferation of cancer cells can be enhanced by the activation of metabolic pathways. However, these results also hint that there is metabolic preference depending on cancer types, which underlies the heterogeneity of cancer.

### Meta co-expression analysis results

**Fig. 2:**
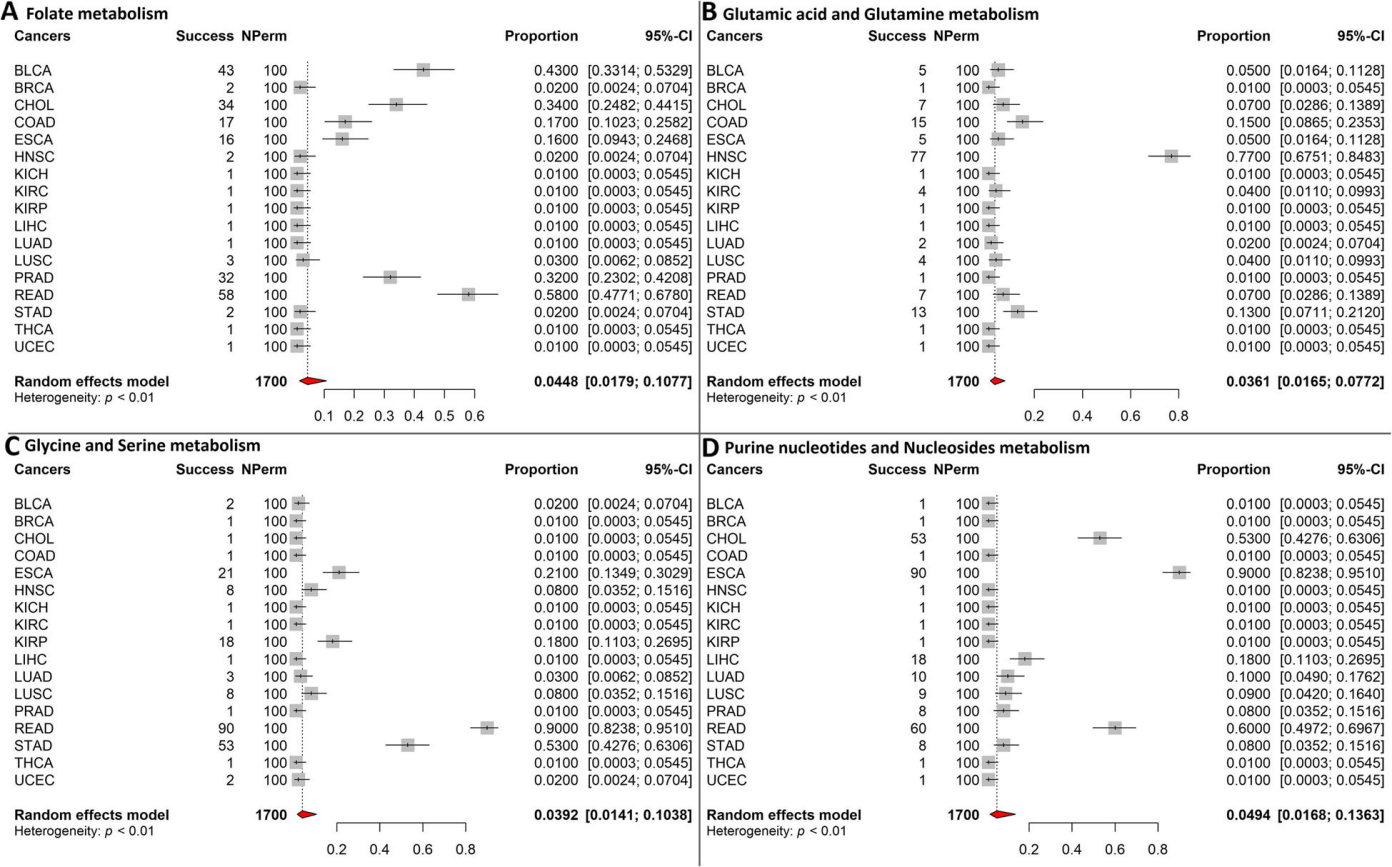
Significant meta co-expression analysis results. **a**. Forest plot for folate metabolism pathway. **b**. Forest plot for glutamic acid and glutamine metabolism pathway. **c**. Forest plot for glycine and serine metabolism pathway. **d**. Forest plot for purine nucleotides and nucleotides metabolism pathway

From a different angle, we dissected metabolic pathway gene expression by contrasting tumor samples and normal samples. Of all 33 TCGA cancer types, only 17 have gene expression data on sufficient normal tissues (*n* > 9). Meta differential co-expression analysis was conducted for each metabolic pathway, with 17 cancer types regarded as multiple datasets. Of the 29 metabolic pathways tested, four showed significant co-expression difference. They are folate metabolism, glutamic acid and glutamine metabolism, glycine and serine metabolism, and purine nucleotide metabolism. Folate is an important member of vitamin B complex and it is essential for cell division and homeostasis. Folate metabolism showed significant co-expression disturbance in 11 of the 17 cancer types, thus resulting in a meta *p*-value of 0.045 (Fig. 2a). Glutamate is a key compound in cellular metabolism, responsible for the biosynthesis of nucleic acid and proteins. Glutamic acid and glutamine metabolism pathway showed significant co-expression disturbance in 12 cancer types, resulting in a meta *p*-value of 0.036 (Fig. 2b). Glycine and serine metabolism is also a vital passage for the biosynthesis of nucleic acids, proteins and lipids. Its pathway showed significant co-expression disturbance in 12 cancer types, producing a meta *p*-value of 0.039 (Fig. 2c). Purine nucleotides metabolism is responsible for synthesizing purine nucleotides and is involved in a plethora of cellular functions. Its pathway showed significant co-expression disturbance in nine cancer types, translating to a meta p-value of 0.049 (Fig. 2d). Collectively, the significant results of these four pathways show that the co-expression network of important DNA synthesizing metabolic pathways have been disturbed during tumorigenesis.

### Differential expression analysis

**Fig. 3:**
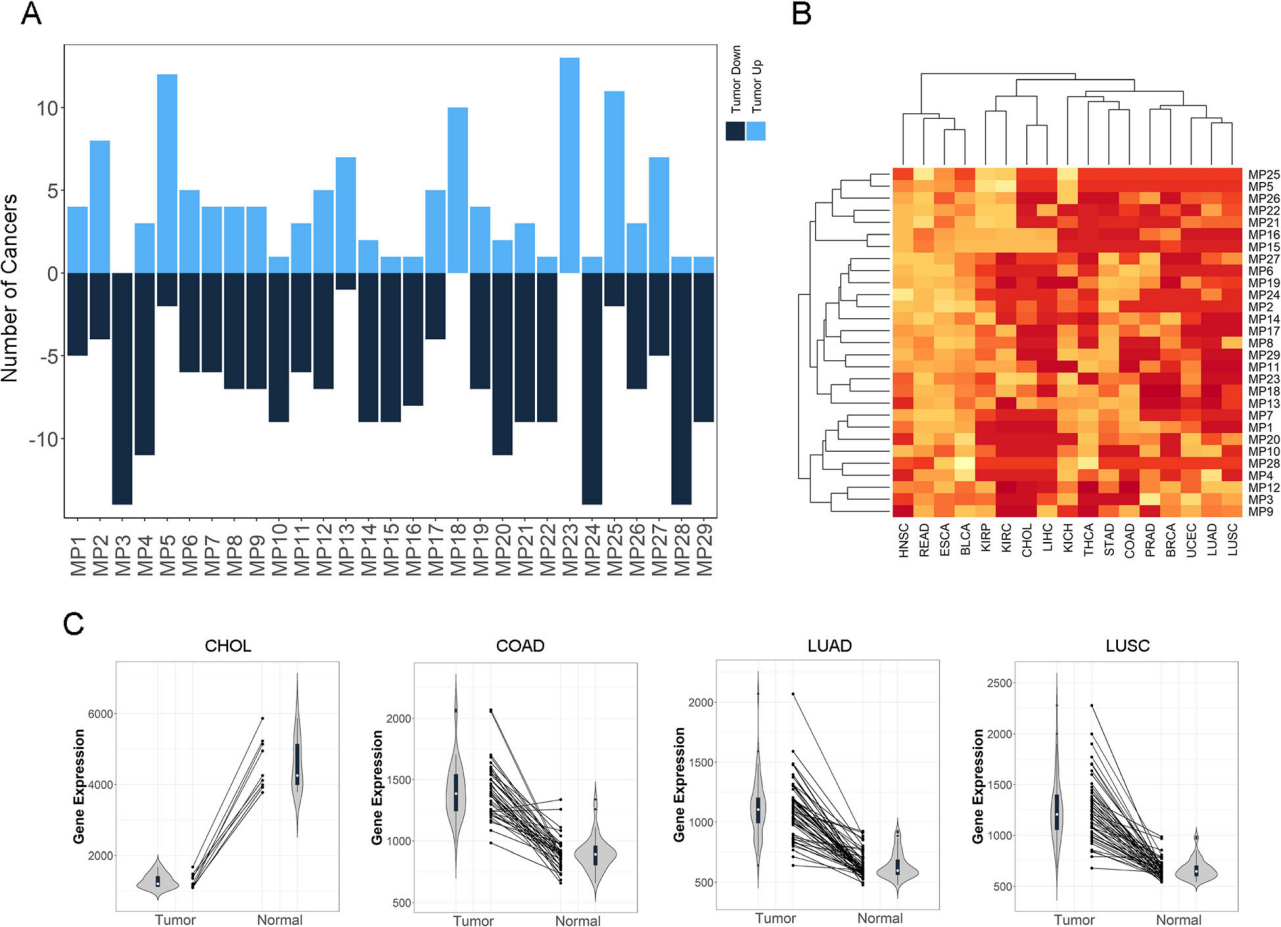
Differential expression analysis results between paired tumor and normal samples. A total of 17 cancer types and 29 metabolic pathways were tested. a. Barplot denotes the number of cancers in which each metabolic pathway showed significant expression changes (up or down regulation). **b**. Heatmap and cluster analysis results using adjust p-value and Euclidian distance. **c**. Patient-wise average gene expression of folate metabolism pathway. Violin plots display the distribution of patient-wise average expression; line segments connect paired average expression values of the same patients

In a comparison setting between tumor tissues and matched normal tissues, we examined the difference in overall expression magnitude of metabolic pathways. Again, the same 17 cancer types with sufficient normal RNA-seq data were enrolled in this analysis. However, we only used paired tumor and normal sample for this analysis. Of all possible 493 (49×17) cancer-pathway combinations, 328 (66%) showed significantly different expression post multiple test adjustment, signaling significant expression alteration between tumor and normal samples for the metabolic pathways tested. Of these 328 dysregulated cancer-pathway combinations, 126 had higher expression in tumor samples and 202 had higher expression in normal samples (Fig. 3a, Supplementary Table S5). Some metabolic pathways showed strong directional preference. For example, MP3 (arginine and proline metabolism) was significantly dysregulated in 14 cancer types, and all of them showed downregulation in tumor. MP23 (purine nucleotides metabolism) was significantly dysregulated in 13 cancer types, all of which showed upregulation in tumor. Cluster and heatmap analysis on the adjusted *p*-values show that lung adenocarcinoma (LUAD) and lung squamous cell carcinoma (LUSC) formed a unique cluster branch (Fig. 3b). Nearly all of the 29 metabolic pathways were significantly dysregulated between paired tumor and normal samples in these two cancer types. Furthermore, we plotted the average gene expression of folate metabolism pathway for individual subjects of four cancers as examples (Fig. 3c). In LUAD and LUSC, a universal upregulation of folate metabolism pathway was observed between all paired tumor and normal samples. In colon adenocarcinoma (COAD), majority of the tumor-normal pairs showed upregulation in tumors. In cholangiocarcinoma (CHOL), all tumor-normal pairs showed downregulation of folate metabolism pathway in tumor. The inconsistent directionality of metabolic pathway expression changes hints at the cancer heterogeneity.

## Discussion

Tumor cells reprogram metabolism to sustain cell proliferation. This is designated as the Warburg effect, which refers to the fact that tumor cells prefer metabolism via glycolysis rather than the efficient oxidative phosphorylation pathway. A myriad of cancer metabolism studies has proved that cancer cells require alternative activation of metabolic pathways to obtain the necessary energy source for cell growth. The direct relation between metabolic pathway expression and cancer prognosis has not been established. Since a metabolic pathway includes multiple genes, survival analyses evaluating a pathway as a single entity has been difficult. Using a newly developed method AESA, we constructed a composite gene expression score to represent the expression level of a single pathway and thus managed to conduct OS and DSS analyses based on these scores. Overall, 166 and 170 cancer-pathway combinations were found to be significantly associated with survival for OS and DSS respectively. All significant results show that higher expression of metabolic pathways resulted in worse survival outcomes. These results strongly suggest that upregulation of metabolic pathway genes reinforced/activated select metabolic pathways, which translated to poor prognosis. Not to confuse with our analysis between tumor and normal, the survival analysis was conducted entirely using tumor samples. As we have shown, the gene expression of metabolic pathways was not always higher in tumor than normal. Additionally, the survival analysis results also show that cancer types have varied survival sensitivity toward metabolic pathway expression change. LGG and KIRC had strong survival associations with all tested metabolic pathways, while some cancer types had no survival association at all.

Our second analysis dealt with the co-expression disturbance of metabolic pathways between tumor and normal samples. This analysis identified four metabolic pathways that showed repetitive co-expression disturbance across multiple cancers. They are folate metabolism, glutamic acid and glutamine metabolism, glycine and serine metabolism, and purine nucleotide metabolism. Folate, also known as vitamin B9, is essential for DNA and RNA synthesis and maintenance of methylation reaction in cells. Folate metabolism is pivotal for cell replication and survival. Interruption of folate metabolism pathway may produce substantial toxic effect on cell division process, the key to tumorigenesis. Inhibition of folate metabolism pathway has been used in cancer treatment [30]. It has been long documented that tumor cells are avid glutamine consumers [31]. Glutamine also plays a crucial role in the uptake of essential amino acid and in maintaining the activation of target of rapamycin (TOR) kinase, which is the central component of the well-known cancer pathway mTOR [32]. Glycine and serine metabolism has been considered as the central hub of cancer metabolism. Serine biosynthesis can be used by glycolysis pathways, which is activated by cancer cells to sustain anabolism. The purine nucleotides metabolism is another important component in the whole DNA synthesis process. Purine analogues also known as antimetabolites can be used for cancer treatment because they have a similar chemical structure to purine. Masquerading as purine, these analogues interfere with DNA synthesis, preventing tumor cells from further dividing [33]. These four metabolic pathways with significant co-expression differences between tumor and normal samples are connected intricately by the complicated one carbon metabolic network, which is centered on folate. Serine is served as a donor to folate one carbon unit. Glycine is an important precursor for purine biosynthesis. Glycine can provide carbon units for one carbon metabolism. Glycine can be converted from serine by glycolysis, which is stimulated by glutamine.

Our last analysis is the differential gene expression analysis of each metabolic pathway between paired tumor and normal samples. It is important to distinguish this analysis from the aforementioned survival analysis, which was based on only tumor gene expression data. In the survival analysis, we observed universal poor prognosis for higher expression of metabolic pathways. However, in the differential expression analysis, we observed more down regulation of metabolic pathway expression in tumor compared to normal. Most metabolites are nutrients for cellular functions. Healthy metabolisms are essential for normal cell growth. Deficiency in certain metabolites can increase cancer risk. For example, chronicle insufficient intake of folate may increase risk of many cancer types [34]. However, upon tumorigenesis, antifolate drug can be prescribed for treatment. Metabolism is essential for all cell survival including both normal and tumor cells. The fundamental difference between metabolism in tumor and normal cells is how metabolisms are activated and utilized.

Our genomic data source TCGA has several limitations. The OS data in TCGA was subjected to immortal time bias [21], the bias towards long survival because of the time gap between the diagnosis time-point and the enrollment time-point. This unavoidable time gap indirectly enforces the study to preferentially enroll patients with longer survival time, thus favoring survival. The DSS data, by contrast, is a more robust measure of a disease’s real impact. However, DSS data tends to have less statistical power due to fewer outcome events. Additionally, TCGA had limited RNA-seq data on adjacent normal tissues. On average, each cancer type has 0 to 10% adjacent normal tissue samples for all subjects enrolled. This limited our analyses to merely 17 cancer types when normal samples were required.

## Conclusion

Our study discovered several important findings. The most novel finding is that higher metabolic pathway expression corresponds to worse survival. The differential co-expression and differential expression analyses demonstrated the disruption of metabolic pathway gene expression between tumor and normal tissues. In summary, these results show that heterogeneous tumor types bear varied sensitivity to metabolic pathway expression changes.

## Supplementary Information

**Additional file 1.**

**Additional file 2.**

**Additional file 3.**

**Additional file 4.**

**Additional file 5.**

## Data Availability

All data used in this manuscript were from public data repositories. Pathway information was obtained form Pathway Commons http://www.pathwaycommons.org/. TCGA data were obtained from Genomic Data Commons Data Portal https://portal.gdc.cancer.gov/.

## Abbreviations

AESA: Advanced expression survival analysis
ACC: Adrenocortical Carcinoma
BLCA: Bladder Urothelial Carcinoma
BRCA: Breast Invasive Carcinoma
CESC: Cervical Squamous Cell Carcinoma and Endocervical Adenocarcinoma
CGES: Composite gene expression score
CHOL: Cholangiocarcinoma
COAD: Colon Adenocarcinoma
DLBC: Lymphoid Neoplasm Diffuse Large B-cell Lymphoma
DSS: Disease specific survival
ESCA: Esophageal Carcinoma
GBM: Glioblastoma Multiforme
GSNCA: Gene Sets Net Correlations Analysis
HNSC: Head and Neck Squamous Cell Carcinoma
KICH: Kidney Chromophobe
KIRC: Kidney Renal Clear Cell Carcinoma
KIRP: Kidney Renal Papillary Cell Carcinoma
LAML: Acute Myeloid Leukemia
LGG: Brain Lower Grade Glioma
LIHC: Liver Hepatocellular Carcinoma
LUAD: Lung Adenocarcinoma
LUSC: Lung Squamous Cell Carcinoma
MESO: Mesothelioma
OS: Overall survival
OV: Ovarian Serous Cystadenocarcinoma
PAAD: Pancreatic Adenocarcinoma
PCPG: Pheochromocytoma and Paraganglioma
PRAD: Prostate Adenocarcinoma
READ: Rectum Adenocarcinoma
SARC: Sarcoma
SKCM: Skin Cutaneous Melanoma
STAD: Stomach Adenocarcinoma
TCGA: The cancer genome atlas
TGCT: Testicular Germ Cell Tumors
THCA: Thyroid Carcinoma
THYM: Thymoma
UCEC: Uterine Corpus Endometrial Carcinoma
UCS: Uterine Carcinosarcoma
UVM: Uveal Melanoma
